# Human iPSC-derived neurons reveal NMDAR-independent dysfunction following HIV-associated insults

**DOI:** 10.3389/fnmol.2023.1353562

**Published:** 2024-01-29

**Authors:** Alexander Starr, Emily Nickoloff-Bybel, Razan Abedalthaqafi, Naela Albloushi, Kelly L. Jordan-Sciutto

**Affiliations:** Department of Oral Medicine, School of Dental Medicine, University of Pennsylvania, Philadelphia, PA, United States

**Keywords:** iPSC, neuron, HIV, microglia, NMDA, antiretroviral therapy

## Abstract

The central nervous system encounters a number of challenges following HIV infection, leading to increased risk for a collection of neurocognitive symptoms clinically classified as HIV-associated neurocognitive disorders (HAND). Studies attempting to identify causal mechanisms and potential therapeutic interventions have historically relied on primary rodent neurons, but a number of recent reports take advantage of iPSC-derived neurons in order to study these mechanisms in a readily reproducible, human model. We found that iPSC-derived neurons differentiated via an inducible neurogenin-2 transcription factor were resistant to gross toxicity from a number of HIV-associated insults previously reported to be toxic in rodent models, including HIV-infected myeloid cell supernatants and the integrase inhibitor antiretroviral drug, elvitegravir. Further examination of these cultures revealed robust resistance to NMDA receptor-mediated toxicity. We then performed a comparative analysis of iPSC neurons exposed to integrase inhibitors and activated microglial supernatants to study sub-cytotoxic alterations in micro electrode array (MEA)-measured neuronal activity and gene expression, identifying extracellular matrix interaction/morphogenesis as the most consistently altered pathways across HIV-associated insults. These findings illustrate that HIV-associated insults dysregulate human neuronal activity and organization even in the absence of gross NMDA-mediated neurotoxicity, which has important implications on the effects of these insults in neurodevelopment and on the interpretation of primary vs. iPSC *in vitro* neuronal studies.

## 1 Introduction

People living with human immunodeficiency virus-1 (HIV) are at increased risk of neurocognitive dysfunction despite effective suppression of viral load, transmissibility, and immune deficiency via combined antiretroviral therapy (ART) (Saylor et al., 2016; Farhadian et al., 2017; Amusan et al., 2020). HIV associated neurocognitive disorders (HAND) effect a broad array of central nervous system (CNS) functions, including learning, memory, cognition, mood regulation, and motor function, and their putative causal mechanisms are equally varied (Cysique et al., 2004; Sakamoto et al., 2013). Some are intrinsic to the virus, including the legacy effects of acute infection (Alakkas et al., 2019; Qu et al., 2022; Yoshihara et al., 2022), persistent low-level replication of virions and viral components in reservoirs (Cochrane et al., 2022; Kreider and Bar, 2022; Gabuzda et al., 2023; Tang et al., 2023), and chronically elevated levels of inflammatory cytokines, glutamate, and reactive oxygen species in the CNS and periphery (Gelman, 2015; McGuire et al., 2015; Ambrosius et al., 2019; Gorska and Eugenin, 2020; Gruenewald et al., 2020). Common external risk factors include substance abuse, comorbidities including opportunistic coinfections, and even toxicity from ART itself (Hauser et al., 2007; Akay et al., 2014; Gill and Kolson, 2014; Hidalgo et al., 2015; De Benedetto et al., 2020; Santos et al., 2020). Despite the wealth of evidence for these mechanisms provided by clinical observations and in animal models, effective treatments to decrease HAND risk or progression have not yet been identified (Kolson, 2022).

Modeling CNS HIV-associated toxicity is challenging due to a number of factors, including the restriction of HIV replication to human CD4^+^ cells and the inherent challenge of studying human neurons *in vitro*, which are post-mitotic and rarely available from primary resections. Previous HIV-related mechanistic work in neurons was largely restricted to either simian immunodeficiency virus (SIV) models, transgenic or humanized rodent models, cross-species exposure of primary rodent neurons to HIV insults generated in human cells, or neuroblastoma-based immortalized neuron-like cell lines (Pappas et al., 1998; Gaskill et al., 2005; Wallace et al., 2007; Jacobs et al., 2019; Nedelcovych et al., 2019; Garcia-Mesa et al., 2020). Despite the vast amount of insights these models have provided into the impact of HIV in the CNS, there remain limitations on the interpretation of the data reported due to the lack of effectively replicating HIV, cross-species immune reactions, and limited recapitulation of neuronal life cycles and activity. It is possible that the challenge of translating mechanisms uncovered in these models into effective therapies is increased by these limitations.

To that end, recent innovations in cell modeling led to the development of induced pluripotent stem cells (iPSCs), adult somatic cells—frequently fibroblasts or peripheral blood mononuclear cells (PBMCs)—that are reprogrammed into pluripotent stem cells using transcription factors (Takahashi and Yamanaka, 2006). Once reprogrammed, these cells can be further differentiated into a wide variety of cells, including those of the CNS. Protocols for differentiation of iPSCs into neurons, astrocytes, and microglia are well-validated and commercially available (Dolmetsch and Geschwind, 2011; Wang et al., 2013, 2017; Ryan et al., 2020). There are also several reported protocols for generation of iPSC-derived oligodendrocytes and precursors, although they are more limited in their commercial availability (McCaughey-Chapman and Connor, 2023). In addition to direct differentiation of monocultures, co-cultures and spontaneous differentiation/maturation of iPSCs in three dimensional organoids provide opportunities to observe cell-cell interactions and supra-cellular structures (Qian et al., 2016; Engle et al., 2018; Bauersachs et al., 2022). Within these models, differentiation is usually accomplished either by supplementation of small molecules and growth factors and/or inducible activation of transcription factors that determine cellular fate.

We previously described an iPSC-driven neuronal model that uses a doxycycline-inducible Neurogenin 2 (NGN2) cassette inserted into a safe harbor locus to rapidly generate glutamatergic cortical neurons (Ryan et al., 2020). When these neurons were co-cultured alongside iPSC-derived microglia (iMg) and astrocytes (iAst), enhanced integrated stress response and inflammation were observed when exposed to HIV and the reverse transcriptase inhibitor, efavirenz; however, gross neuronal toxicity was not reported, despite being repeatedly observed in primary rodent models exposed to similar insults (Wang et al., 2007; Zyskind et al., 2015; Stern et al., 2018a,b). It is not clear whether this was due to the presence of other cell types, the functional maturity of the iPSC-derived neurons, or species differences between rodents and humans.

Thus, we endeavored to better understand HIV-associated toxicity in human neurons using integrated, inducible, isogenic (i^3^) neurons (Wang et al., 2017; Fernandopulle et al., 2018). Similar to our previous model, i^3^ neurons rely on a doxycycline inducible neurogenin 2 (NGN2) to achieve high purity populations of glutamatergic cortical neurons. In order to separate out neuron-specific responses, i^3^ neurons were monoculture and exposed to a number of HIV-associated insults, including supernatants from HIV-infected or lipopolysaccharide (LPS)-treated human monocyte-derived macrophages (MDM) and iMg and the integrase strand transfer inhibitors (INSTIs), elvitegravir and raltegravir. We found that i^3^ neurons in monoculture remain resistant to cell death when exposed to stimuli that were reported to be neurotoxic in primary rodent neurons, perhaps mediated by their demonstrated resistance to *N*-methyl-D-aspartate (NMDA) receptor-mediated toxicity; however, both convergent and divergent alterations in cell morphology, spontaneous neural activity, and transcriptional pathways were identified.

## 2 Materials and methods

### 2.1 iPSC-derived neuron differentiation and maturation

Human i^3^ cortical neurons were generated as previously described (Wang et al., 2017; Fernandopulle et al., 2018). In brief, human iPSCs reprogrammed from a wild type male containing a doxycycline-inducible NGN2 integrated into the AAVS1 safe harbor locus were cultured on matrigel-coated (Corning) plates in supplemented Essential 8 medium (Gibco). Once iPSC cultures reached confluence, they were split and induced toward neuronal differentiation with doxycycline-containing induction media (DMEM/F12-Gibco, N2, and NEAA-Invitrogen) for 3 days. On DIV 3, induced neurons were lifted and plated on the final experimental plates for maturation (PLO/Laminin-coated Corning Cell-Bind plates or PEI/Laminin-coated Axion CytoView MEA plates). Neurons were plated at a density of 1.2 × 10^5^ cells per ml for imaging experiment and 2 × 10^5^ cells per ml for MEA experiments. Cells were matured in cortical maturation media (BrainPhys-Stem Cell Technologies, B-27-Gibco, BDNF, NT3, and laminin) for an additional 14–18 days prior to experiment initiation.

### 2.2 MDM and iMg supernatant generation and supplementation

Monocyte derived macrophages (MDMs) were differentiated as previously described. In brief, primary human monocytes isolated by the Penn Human Immunology Core were plated at 2.4 × 10^5^ cells per ml and differentiated in DMEM containing human serum and M-CSF for 7 days. For HIV-Jago experiments, MDMs were differentiated using GM-CSF. iMg were generated from 9 day old common myeloid progenitors provided by the CHOP Stem Cell Core and differentiated in microglia for 11 days in RPMI (HyClone) containing FBS, M-CSF, IL-34, and TGF-β, as previously described (Ryan et al., 2020). Fully differentiated cells were treated with HIV-ADA at a concentration of 1 ng/ml HIV p24 (day post-infection 0, DPI 0). After 24 h, HIV-containing media was removed and supernatants were collected every 48 h. Experimental supernatants were collected on DPI 9. For LPS + ATP samples, uninfected cultures were treated with 1 ng/ml E. coli LPS for 23.5 h beginning on DPI 8, with a 30 min addition of 2.5 mM ATP prior to supernatant collection. i^3^ neurons were treated with supernatants at a ratio of 1:4 in cortical maturation media beginning on neuronal DIV 19 and maintained for 4 days.

### 2.3 NMDA treatment

DIV 21 i^3^ neurons were treated with vehicle (water) or NMDA (400 or 800 μM) for 24 h, at which time they were fixed for staining.

### 2.4 Integrase inhibitor preparation and treatment

Elvitegravir and raltegravir were resuspended in dimethyl sulfoxide (DMSO, Veh). Working stocks were prepared via serial dilution in DMSO to ensure all doses (0.1, 1, and 10 μM) contained the same amount of DMSO. i^3^ neurons were treated with INSTIs beginning on DIV 18 and maintained for 4 days for viability experiments, and 9 days for MEA experiments. Fifty percent media changes every other day were supplemented with an additional half dose of drug to ensure any removed was replaced, but no drug metabolism was assumed.

### 2.5 Immunofluorescent staining

Neurons were fixed in the culture well with 4% paraformaldehyde for 10 min, permeabilized with 0.1% Triton-X, and blocked with 5% normal goat serum and 0.5% bovine serum albumin in PBS. Primary guinea pig anti-MAP2 antibody (Synaptic Systems #188004) was diluted 1:1000 in block and incubated overnight at 4°C. Secondary goat anti-guinea pig antibody conjugated to FITC (1:200) and 4′,6-diamidino-2-phenylindole, dihydrochloride (DAPI, 1:2500) were incubated at 25°C for 1 h. Stained cells were stored at 4°C in PBS until they were imaged.

### 2.6 Imaging and quantification

Images were captured using a Keyence BZ-X710 fluorescent microscope with a 20X objective using DAPI and FITC filter cubes. The automatic stage was used to capture 3 × 3 grids of images around a set center point in each well. Grids were stitched and merged using the BZ-X analyzer software. Macros assigning fluorescence intensity and size gating were used to define DAPI^+^ nuclei and positive MAP2 staining in control wells. These macros were then applied uniformly to all images from the experiment and counts of (a) MAP2^+^ nuclei and (b) total MAP2^+^ area were exported from the BZ-X analyzer software.

### 2.7 Microelectrode array (MEA) recordings

Microelectrode array recordings were conducted on 48-well Axion CytoView plates using an Axion Maestro Pro MEA with neural and viability modules. Baseline recordings were conducted 24 h prior to initial treatment (neuronal DIV 19 for myeloid cell supernatants and DIV 21 for ART treatments) and recordings were then conducted daily for 4 days for supernatant experiments and at 2, 4, 7, and 9 days post ART treatment. Recordings occurred at 37°C for a minimum of 5 min, 30 s per recording. Viability was recorded prior to spontaneous neuronal activity for each recording. All recordings were batch processed and trimmed to 300 s prior to automated analysis and data output using the Axion Neural Metrics Tool. Metrics which are dependent on well-to-well plating variation–including average resistance per well, number of covered electrodes, and mean firing rate–were normalized within wells to the same metric at the time of baseline recording.

### 2.8 RNA isolation and sequencing

Following the completion of MEA experiments, neurons from 6 replicate wells were lysed in the well using TRIzol (Invitrogen) and pooled RNA was isolated using manufacturer protocols. Purity and concentration of RNA were confirmed using Nanodrop, and Zymo RNA Clean and Concentrator-25 was used on any samples that did not pass initial quality control (260/280 > 2, 260/230 > 1.8, minimum concentration 50 ng/μl). Purified RNA was submitted to Azenta Life Sciences/Genewiz for mRNA enriched library preparation and sequencing via Illumina HiSeq, with an average read depth of approximately 22 million reads per sample. Reads were trimmed using Trimmomatic v.0.36 and mapped to the Homo sapiens GRCh38 reference genome using STAR aligner v.2.5.2b. Raw gene counts were calculated using featureCounts from the Subread package v.1.5.2. These raw counts were read into NOISeq v.2.44.0, low counts were filtered out by minimum 5 counts per million reads (CPM), and normalized by reads per kilobase per million mapped reads (RPKM) (Tarazona et al., 2015). Significantly differentially expressed genes (DEGs) were defined by a cutoff of *q* = 0.9. DEGs were ranked by probability of differential expression and the top 30 were subjected to hierarchical clustering of their normalized counts by average Euclidean distance using Heatmapper (Babicki et al., 2016). All DEGs were subjected to gene enrichment analysis using ShinyGO 0.77 and the top 10 most enriched KEGG pathways with an FDR cutoff of 0.05 were reported (Ge et al., 2020; Kanehisa et al., 2021).

### 2.9 Statistical analyses

For supernatant experiments, all conditions (5 MDM donors, 3 iMg lines) were replicated across three wells per experiment and repeated across 2–3 neuronal differentiations. All NMDA and ART treatments were replicated across three wells per experiment and at least 3 neuronal differentiations. Cell counts and area were assessed using unpaired *t*-tests (2 groups) or one-way ANOVA (3 or more groups). For longitudinal MEA experiments, one-way ANOVA was used to capture changes in neural activity over time, as opposed to assessing individual time points. For ART MEA experiments, all doses were individually compared to Veh using one-way ANOVA with Dunnett’s correction for multiple comparisons.

## 3 Results

### 3.1 Human iPSC neuron resistance to HIV-myeloid cell supernatant toxicity

**FIGURE 1:**
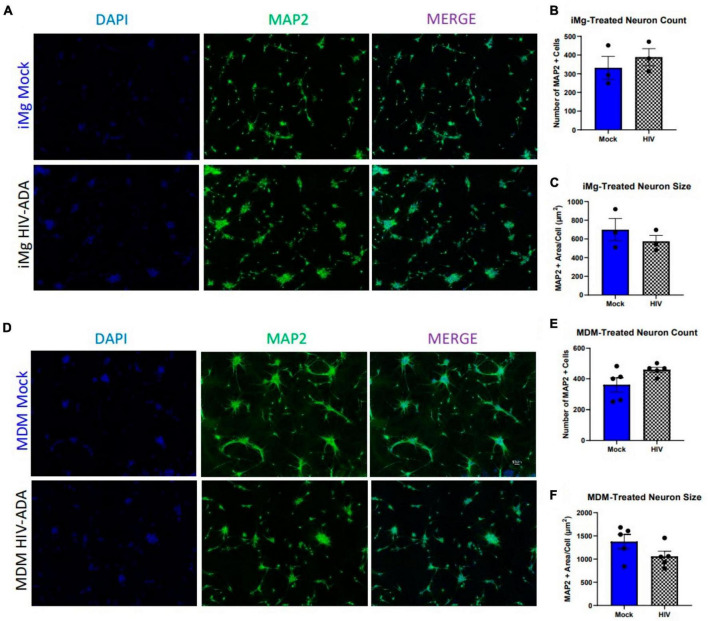
Human i^3^ cortical neurons are resistant to cytotoxicity from HIV-ADA-infected myeloid cell supernatants. **(A)** Representative images of i^3^ neurons following 96-h exposure to HIV-iMg supernatants (*n* = 3 donors, mean ± s.e.m.) and **(D)** HIV-MDM supernatants (*n* = 5 donors, mean ± s.e.m.) **(B,E)** Quantification of average number of MAP2^+^ cells and **(C,F)** average MAP2^+^ area per cell.

Until recently, rodent models have been the primary source of our understanding of HIV-associated neuropathology *in vitro* (Cross et al., 2011; Tovar et al., 2013; Stern et al., 2018a,b; Lopez Lloreda et al., 2022). We sought to expand upon these findings and validate them in a human iPSC-derived cortical neuron model (i^3^ neurons) that achieves neuronal differentiation by inducible expression of integrated *NGN2* transcription factor expression (Wang et al., 2017; Fernandopulle et al., 2018). Cortical, in this case, does not refer to their derivation from a specific anatomical region, but gene expression of markers, including *CUX1* and *SLC17A7*, which are commonly found in primary cortical glutamatergic neurons (Wang et al., 2017). These and other iPSC-derived models offer an attractive alternative/supplement to primary rodent models due to their human origin, high purity, and the ability to differentiate and even co-culture multiple cell types, including neuronal subtypes and glia, from the same source in a defined ratio (Qian et al., 2016; Ryan et al., 2020). The latter advantage is particularly useful for modeling diseases with genetic variations that are difficult to define or reproduce in animal models, as isogenic correction can provide within-individual evaluation of genetic contributions (Dolmetsch and Geschwind, 2011; Engle et al., 2018). In order to begin our characterization of this neuronal model, we first sought to replicate previous findings in rodent and human primary cultures which demonstrated neuronal death upon supplementation with supernatants from HIV-infected cells (Jiang et al., 2001; Chen et al., 2002; Wang et al., 2007; Cross et al., 2011; Colacurcio et al., 2013; Stern et al., 2018a). Importantly, the sources of these supernatants and the nature of the cultures vary widely, including a variety of HIV strains and concentrations applied to peripheral blood mononuclear cells (PBMCs), macrophages, or microglia as the source of supernatants applied to primary neurons, neuronal cell lines, and a neuro-glial combined cultures. We began with a well-established model, using primary human monocytes differentiated in macrophages and infected with the macrophage-tropic molecular clone, HIV-ADA (Jiang et al., 2001). Initial dose (1 ng/ml) and day of supernatant collection were chosen to ensure productive infection without macrophage toxicity. Despite robust infection in these macrophages, supplementation of i^3^ neurons with HIV-MDM supernatants did not reduce the number of MAP2^+^ cells relative to mock supernatants, even at a supplementation ratio of 1:4 (Figures 1A, B). In addition, there was no reduction in the MAP2^+^ area per cell (Figure 1C), indicating there were no significant morphological alterations or loss of neuronal processes, which frequently precede neuronal death (Kim et al., 2008; You et al., 2023). To ensure our imaging paradigm was not failing to capture cell death, we also analyzed culture supernatants for lactate dehydrogenase (LDH) release, which confirmed there was no increase in neurotoxicity in HIV supernatant cultures (Supplementary Figure 2C). This was also true for iMg, which are reliably infected but produced less virus with the same infection paradigm (Figures 1D–F).

Given evidence that toxicity observed in previous models using similar paradigms was glutamate dependent, we measured glutamate in these supernatants via Amplex Red fluorescent assay (Jiang et al., 2001; Chen et al., 2002; O’Donnell et al., 2006; Porcheray et al., 2006). While we did observe slight increases in glutamate using both models, the increase was not statistically significant and, in the case of MDMs, was well below the glutamate levels in fresh media (Supplementary Figures 2A, B). We then repeated these experiments using MDM supernatants generated using our previously published model, which uses a higher dose of the macrophage-tropic HIV-Jago strain, maintains infection for 15 days, and leads to increases in both glutamate and primary rat neuron toxicity (Lopez Lloreda et al., 2022). This paradigm also failed to induce neuronal loss in human i^3^ neurons (Supplementary Figures 1A–C), indicating that the degree of viral replication and glutamate production were not likely limitations of our initial observations. Importantly, these measurements report glutamate in bulk media; thus, any alterations in local glutamate metabolism at the synapse, where glutamate concentrations are typically much higher than in the surrounding CSF, would not have been observed with this assay.

### 3.2 NMDAR-independent toxicity in iPSC neurons

**FIGURE 2:**
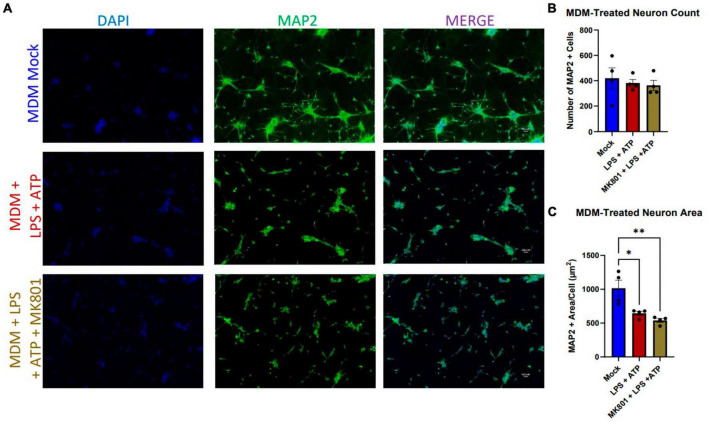
LPS + ATP-treated myeloid cell supernatants reduce neuronal area in an NMDAR-independent manor. **(A)** Representative images of i^3^ neurons following 96-h exposure to LPS-MDM supernatants (*n* = 4 donors, mean ± s.e.m., One-way ANOVA, *p < 0.05, **p < 0.01). **(B)** Quantification of average number of MAP2^+^ cells and **(C)** average MAP2^+^ area per cell.

We were then curious whether the resistance to neurotoxicity observed across a variety of HIV supernatant paradigms in i^3^ neurons was a general resistance to the activated myeloid cell secretome, or specific to HIV. Previous studies have used bacterial lipopolysaccharide (LPS) to activate myeloid cells and induce neurotoxicity, especially in the context of HIV infection (Jiang et al., 2001; Gajanayaka et al., 2017; Jerebtsova et al., 2020). Beyond its usefulness as a known immune activator, LPS is clinically relevant in the context of HIV infection, where gut microbial translocation leads to increased levels of plasma LPS (Nottet et al., 1996; Kumar et al., 2016). While increased levels of LPS have not been observed in cerebrospinal fluid (CSF), blood-brain barrier disruption and increased peripheral immune cell infiltration are known hallmarks of HIV infection, and these peripheral cells will be exposed to plasma LPS prior to infiltration (Persidsky et al., 1999; Chaudhuri et al., 2008; Jiang et al., 2021). Thus, we exposed differentiated MDMs to E. coli LPS (1 ng/mL) for 23.5 h, followed by a 30 min addition of 2.5 mM ATP to induce robust immune response and ensure inflammasome activation (Walsh et al., 2014). Exposure of i^3^ neurons to these supernatants did not result in neuronal loss, but did significantly reduce neuronal area (Figures 2A–C). Given previous evidence that LPS toxicity is glutamate mediated and blockable by the non-competitive NMDAR receptor antagonist MK801, we attempted to prevent this morphological damage using a 1 h pre-treatment with 10 μM MK801. This was not effective (Figure 2C), suggesting that LPS caused NMDAR-agnostic damage in i^3^ neurons. This differed from previous reports, where MK801 not only prevented toxicity, but accounted for a complete rescue of observed toxicity (Lipton, 1994; Jiang et al., 2001; O’Donnell et al., 2006).

**FIGURE 3:**
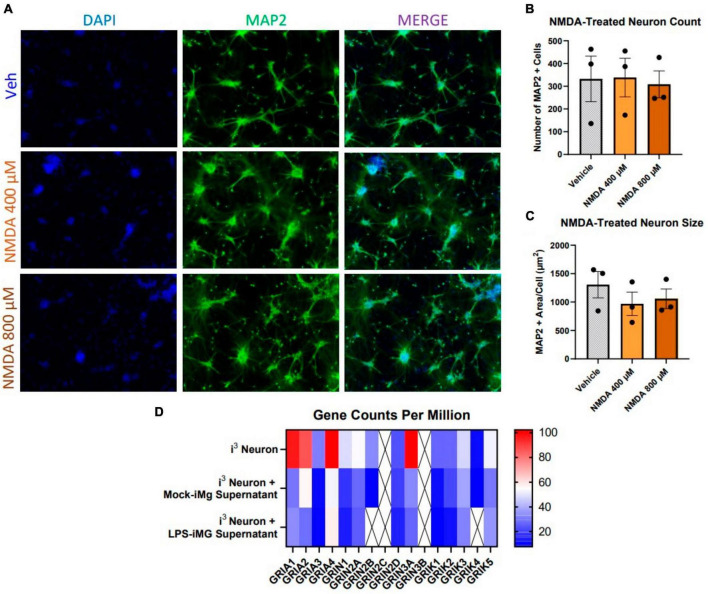
Human i^3^ neurons are resistant to NMDA-mediated cytotoxicity. **(A)** Representative images of i^3^ neurons following 24-h exposure to NMDA (*n* = 3 neuronal differentiations, mean ± s.e.m.). **(B)** Quantification of average number of MAP2^+^ cells and **(C)** average MAP2^+^ area per cell. **(D)** RNA-Seq read counts per million of glutamate receptor subunit genes expressed by i^3^ neurons with and without iMg supernatant supplement (crossed out box = not detected, minimum CPM cutoff = 5).

Our accumulating evidence suggested that i^3^ neurons were resistant to glutamate-mediated toxicity, as did a number of previous reports in other human, stem-cell derived neuronal models (Sattler et al., 1997; Dolmetsch and Geschwind, 2011; Donnelly et al., 2013; Gupta et al., 2013; Bauersachs et al., 2022), which suggest that glutamate was toxic to iPSC-derived neurons only at concentrations 3x those in primary mouse cultures. In order to address this question directly, we initially exposed neurons to 3, 10, and 30 μM NMDA for 1 h and fixed 23 h later, which did not result in cell loss or reduction in area (data not shown). Given the reports of NMDA-resistance cited above, we then applied supraphysiological doses of 400 and 800 μM and maintained this treatment for 24 h prior to fixation. Even these elevated doses and prolonged exposure did not reduce neuron count or size (Figures 3A–C).

This observation suggested i^3^ neurons might express low levels of NMDAR receptor genes or express subunits in a ratio which was not optimal for classical NMDAR activity. Using RNA-seq, we assayed gene expression for all subunits of glutamate receptors, including AMPA-receptors (*GRIA*), NMDA receptors (*GRIN*), and kainite receptors (*GRIK*), in i^3^ neuron cultures both with and without iMg supernatant supplementation (Figure 3D). In supernatant-free cultures, expression of AMPAR transcripts was considerably higher than other glutamate receptors. The two most highly expressed NMDAR transcripts were *GRIN2A* and *GRIN3A*. *GRIN3A*, a non-conventional NMDAR subunit that actually counters classical NMDAR activity, is primarily expressed in early development, and its elevated expression reflects possible developmental immaturity of iPSC neurons (Kehoe et al., 2013; Crawley et al., 2022).

Supplementation with either mock or LPS-treated iMg supernatants similarly reduced expression of all nearly all glutamate receptor transcripts (Figure 3D), including AMPARs. Reduced AMPAR expression could further reduce the ability of NMDARs to respond to glutamate in myeloid cell supernatants due to a lack of activating depolarization via AMPA receptors. The combination of resistance to glutamate and NMDA-mediated toxicity across multiple paradigms along with transcriptional evidence of reduced expression of classically functional glutamate receptors indicated that i^3^ neurons could be useful for examining NMDAR-independent pathogenicity.

### 3.3 Comparative analysis of integrase inhibitor-mediated dysfunction in iPSC neurons

**FIGURE 4:**
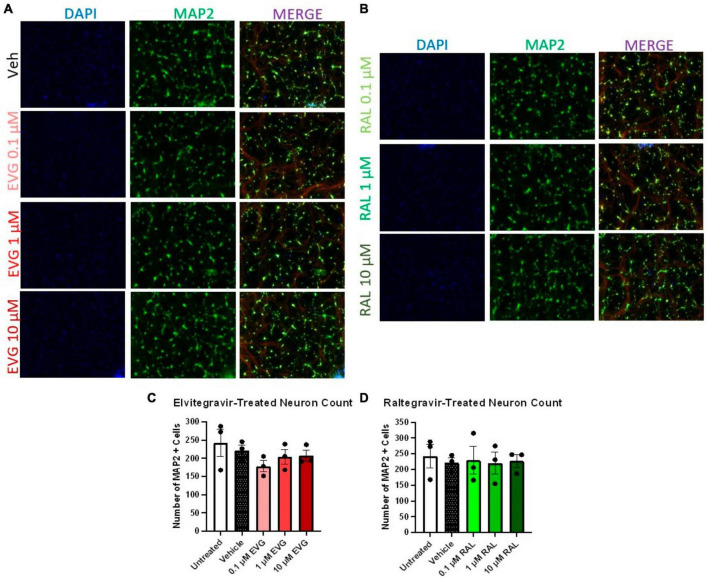
Integrase inhibitors elvitegravir and raltegravir are not cytotoxic to i^3^ neurons. **(A)** Representative images of i^3^ neurons following 9 days of exposure to 3 doses of elvitegravir (EVG) or **(B)** raltegravir (RAL, *n* = 3 neuronal differentiations mean ± s.e.m). **(C,D)** Quantification of average number of MAP2^+^ cells. Staining for Beta III tubulin (Red) is present in merged images but is not part of the analysis.

Having established the resistance of iPSC neurons to glutamate-mediated, HIV-associated cell death, we next examined whether integrase inhibitor toxicity translated to the human model. We previously reported that elvitegravir (EVG), but not raltegravir (RAL), was neurotoxic in primary rat neuroglial cultures, and that this toxicity was driven by activation of the integrated stress response (ISR) (Stern et al., 2018b). We therefore hypothesized that i^3^ neurons might undergo gross cytotoxicity in the presence of EVG, but not RAL, especially since this toxicity was not known to depend on NMDARs. We treated neurons with 0.1, 1, and 10 μM doses of both EVG and RAL (roughly corresponding to 0.025, 0.25, and 2.5X adult plasma Cmax) in mixed neuronal cultures for 4 days. At an extended treatment time point of 9 days, no gross toxicity was observed with either EVG or RAL at any dose (Figures 4A–D). While this was consistent with previous reports on raltegravir, the lack of obvious elvitegravir toxicity was not (Blas-Garcia et al., 2014; Smith et al., 2022). Intriguingly, gross loss of MAP2^+^ cells was observed in i^3^ neurons treated with Biktarvy, a combination antiretroviral drug that includes the integrase inhibitor, bictegravir, along with emtricitabine and tenofovir alafenamide (Supplementary Figures 3A, B). This indicates that observation of ART-induced toxicity is possible using the above model and imaging paradigms.

Since dysregulation of spontaneous activity frequently precedes neuronal death, we expanded our analysis of sub-cytotoxic integrase inhibitor insults to include functional analyses using micro-electrode array (MEA). Axion MEA 48-well plates contain electrodes embedded in the plating surface and allowed us to longitudinally monitor viability, spontaneous excitatory activity, and synchronized signaling. Viability was measured using the conductance of a fixed impulse across the electrode, which would be impeded by the cell and report a resistance relative to the amount of intact cellular material covering the electrode. Analyzing the number of covered electrodes provides a correlate of neuronal coverage within the well. Spontaneous activity was measured without any exogenous stimulation, where a spike was recorded as a change in the local field potential over a given electrode that was greater than 6 standard deviations above background and reported as a firing rate of spikes/second. In order to correct for any changes in viability that altered the number of cells able to contribute spontaneous activity, firing rate normalized to viability was also reported. Coordinated activity, both locally and across the well, were reported by counts of bursts (minimum of 5 spikes/500 ms on the same electrode) and network bursts (minimum of 50 spikes/500 ms distributed across at least 35% of active electrodes in the well). These data were recorded over a 9 day period.

**FIGURE 5:**
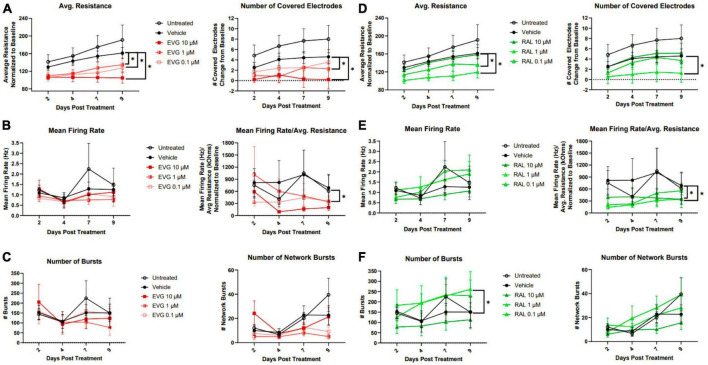
Elvitegravir and raltegravir differentially alter i^3^ neuron spontaneous activity. MEA analysis of i^3^ neurons throughout 9 days of exposure to 3 doses of elvitegravir [EVG, **(A–C)**] or raltegravir [RAL, **(D–F)**, *n* = 3 neuronal differentiations, mean ± s.e.m, one-way ANOVA, *p < 0.05]). **(A,D)** Viability and cell area as measured by conductance. **(B,E)** Spontaneous firing rate and rate normalized to total cellular material. **(C,F)** Synchronous activity as measured by number of local and network burst events.

Compared to vehicle controls, EVG and RAL both reduced average resistance and number of covered electrodes, indicating a loss of electrophysiologically intact cellular material (Figures 5A, D); these effects increased with concentration of EVG, but were only observed with lower concentrations of RAL, a trend which was consistent across other metrics. This loss of neuronal material was surprising given a lack of obvious loss of MAP2 staining in the imaging paradigm, and may reflect a relative insensitivity of MAP2 staining for detection of sub-lethal damage. Initial observations of firing rate did not show an effect of INSTIs, but correction for amount of viable cellular material did show reduced spontaneous activity for both EVG and RAL (Figures 5B, E). The most distinctive metric was burst count, reflective of local, temporal coordination of firing; EVG had no impact on bursts at any concentration, while lower concentrations of RAL significantly increased burst activity, in contrast to their broader effect on spontaneous firing (Figures 5C, F). Network bursts were not affected by either drug.

### 3.4 Converging transcriptional alteration in integrase-inhibitor treated neurons

**FIGURE 6:**
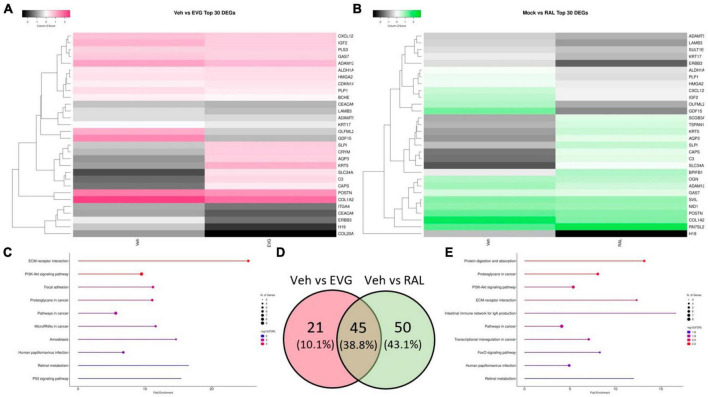
Integrase inhibitors similarly alter i^3^ neuron gene expression. Analysis of significantly differentially expressed genes (DEGs) from RNA-seq on neurons post-MEA analysis. **(A)** Clustered heatmap of log_2_ normalized counts per million for top 30 most significant DEGs between vehicle and 1 μM elvitegravir and **(B)** 1 μM raltegravir. **(C)** Top 10 enriched KEGG pathways containing significant DEGs induced by EVG and **(E)** RAL. **(D)** Venn diagram showing commonalities among significant DEGs induced by integrase inhibitors.

Following the conclusion of MEA recordings, cells were lysed and RNA was extracted for sequencing to identify transcriptional changes that correlated with the observed neuronal activity. In contrast to microglial supernatants, treatment with 1 μM EVG or RAL did not reduce AMPAR or NMDAR transcript expression, indicating that alterations in firing were not simply a reflection of broadly downregulated glutamatergic system (data not shown). One of the gene subsets most commonly downregulated across INSTIs were those associated with extracellular matrix interactions and cell motility, including ADAM metallopeptidases, laminins and collagens, and *C3* and *CXCL12*, classic pro-inflammatory genes with known roles in neural structure and development (Figures 6A–C, E). These observations are consistent with a recent report relating integrase inhibitors’ ability to impair matrix metalloproteinase function with the frequent–yet inconclusive–clinical reports of structural defects in the developing CNS when exposed to INSTIs *in utero* (Xu et al., 2017; Bade et al., 2021; Foster et al., 2022, 2023; Zizioli et al., 2023). The majority of the significant differentially expressed genes altered by EVG were shared with RAL (Figure 6D), and several of the unique RAL genes also involved morphogenesis and TGF-β signaling. The replication of structure-based transcriptional changes across INSTIs is also consistent with the changes in cell coverage observed in MEA assays.

### 3.5 Established neurodegenerative pathways associated with activated microglial supernatants

**FIGURE 7:**
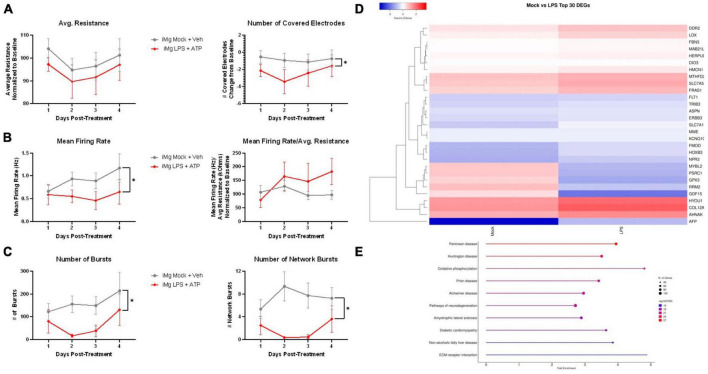
LPS-iMg supernatants alter i^3^ neuron activity and transcription. MEA analysis of i^3^ neurons throughout 4 days of exposure to supernatants from LPS + ATP treated iMg (*n* = 3 neuronal differentiations, mean ± s.e.m, one-way ANOVA with Dunnett’s correction for multiple comparisons, *p < 0.05). **(A)** Viability and cell area as measured by conductance. **(B)** Spontaneous firing rate and rate normalized to total cellular material. **(C)** Synchronous activity as measured by number of local and network burst events. **(D)** Clustered heatmap of log_2_ normalized counts per million for top 30 most significant DEGs between neurons exposed to iMg and LPS-iMg supernatants. **(E)** Top 10 enriched KEGG pathways containing significant DEGs induced by LPS-iMg supernatants.

Having identified convergent neuronal dysfunction in our model using different pharmacological insults, we wished to understand whether these findings also applied to the activated myeloid cell secretome. As with MDMs, iMg were treated with LPS + ATP and their supernatants were added to neuronal cultures for 4 days. MEA conductance assays revealed that while average resistance was the same between mock and LPS-treated supernatants, the number of covered electrodes was reduced, reflecting earlier findings in microscopy assays of reduced cellular area without loss of viable cells (Figure 7A). Reduced firing rate with LPS treatment was not observed after normalization for resistance (Figure 7B). To a much greater extent than in the antiretroviral paradigms, reductions in both local and network-wide synchronized firing were observed (Figure 7C). This was not surprising given the global downregulation of glutamate receptor genes described in these cultures (Figure 3D).

Transcriptomic analysis of these neurons revealed both convergent and divergent pathways between stress paradigms. Extracellular matrix (ECM)-receptor interactions was again one of the top 10 enriched KEGG pathways, as was the case with INSTIs (Figures 7D, E). Although ECM transcripts were again well represented in the top 30 DEGs, the relative enrichment of this pathway was reduced in favor of several neurodegeneration-associated pathways. Examination of the KEGG pathway database suggest this was largely driven by upregulation of genes associated with oxidative stress (*MTHFD2, HYOU1, GPX3*) and downregulation of genes associated with endoplasmic reticulum stress (*HERPUD1*, *HPSA5*, *BIP*, *EIF2AK3*, *ATF4*), mechanisms of neuronal injury reported across multiple neuroHIV-relevant models (Lindl et al., 2007; Akay et al., 2012, 2014; Gill et al., 2014; Gruenewald et al., 2020).

## 4 Discussion

iPSC-derived neuronal models offer several unique advantages beyond their human origin, including the relative ease of generating isogenic controls, the ability to co-culture multiple cell types from the same individual, and the option to use patient-derived cells to study multifactorial risk; however, their developmental state and synaptic immaturity may impair their ability to replicate certain pathogenic mechanisms. In this study, i^3^ neurons were shown to be resistant to a number of NMDAR-associated stimuli, including toxic levels of NMDA itself, calling into question their maturity and homogeneic identity as cortical neurons and highlighting the importance of the anatomical context for functional cell development. This provides not only a challenge, but also an opportunity to identify more subtle mechanisms that may be masked by acute excitotoxicity. While elevated glutamate has been implicated as neurotoxic in *in vitro* models of HAND, clinical studies measuring CSF glutamate levels in people living with HIV are inconclusive regarding both the presence of increased glutamate and any correlation between glutamate level and risk of neurocognitive dysfunction (Espey et al., 1999; Ferrarese et al., 2001; Heaton et al., 2011). Considering HAND and other neurodegenerative conditions are often associated with aging, these more subtle mechanisms may accumulate over time to become major contributors to disease onset and progression. Sub-neurotoxic models can also be useful in neuropsychiatric disorders featuring altered neuronal activity without cell death, and in studies of neurodevelopmental disorders, where affected neurons are not yet synaptically mature (Habela et al., 2016; Sabitha et al., 2021; Saad et al., 2022). One particular sub-neurotoxic phenotype that is relevant in neurodevelopmental disorders is alterations in spine density and morphology, which would not have been captured via our studies of MAP2 morphology and may underlie some the observed changes in neuronal activity. It is also important to note that the functional maturity of all neurons, including iPSC neurons, is affected by their environment. Several of the primary rodent and human studies cited here featured mixed neural-glial cultures, where astrocytes and any remaining microglia can not only facilitate synaptic maturation and function, but can also mediate inflammatory cytokine and glutamate release in response to applied insults, increasing the overall stress on the neurons (Tovar et al., 2013; Castellano et al., 2019; Cheney et al., 2020; Gorska and Eugenin, 2020). This can be recapitulated with iPSCs by co-culturing i^3^ neurons with iPSC-derived astrocytes, microglia, and/or macrophages. In fact, HIV infected iPSC-derived microglia have demonstrated toxicity on iPSC neurons when directly co-cultured (Alvarez-Carbonell et al., 2019). The specificity and purity of iPSC cultures also allows researchers to “build-up” models one cell type at a time to determine which mechanisms are intrinsic to a certain cell, and which require or are enhanced by cell-cell interactions.

The neuronal dysfunction we did observe using this monoculture model had both novel and replicative value. MEA experiments indicated that EVG had a greater impact on cell viability than RAL, as previously reported in rodent models (Stern et al., 2018b; Smith et al., 2022). The shared transcriptomic trend of downregulated ECM-associated genes is the first demonstration in neurons of matrix interaction deficits consistent with those INSTIIs induced in myeloid cells, and may be particularly relevant in a developing neuronal model (Bade et al., 2021; Foster et al., 2023). In LPS-treated microglia supernatant experiments, drastic reduction of coordinated firing was consistent with down regulation of AMPA and NMDA receptor genes, which has been previously reported in CNS cells exposed to LPS-induced inflammation (Pellegrini-Giampietro et al., 1994; Zubareva et al., 2020). Gene expression analysis revealed that LPS-microglia supernatants alter neuronal oxidative and ER stress pathways even in the absence of NDMA-sensitivity, suggesting that NDMAR-mediated calcium influx is not the only way in which activated myeloid cells enhance mitochondrial and ER stress. Taken together, this work validates i^3^ neurons as a useful tool to study both known and novel HIV-associated mechanisms in an NMDAR-independent manner, while also highlighting some of the limitations of iPSC-derived neurons and the need for care in comparing results obtained from different neuronal sources.

## Data availability statement

The datasets presented in this study can be found in online repositories. The names of the repository/repositories and accession number(s) can be found below: https://www.ncbi.nlm.nih.gov/geo/, GSE250609.

## Ethics statement

Ethical approval was not required for the studies on humans in accordance with the local legislation and institutional requirements because only commercially available established cell lines were used. GEO belongs to public databases. The patients involved in the database have obtained ethical approval. Users can download relevant data for free for research and publish relevant articles. Our study is based on open-source data, so there are no ethical issues or other conflicts of interest.

## Author contributions

AS: Conceptualization, Data curation, Formal analysis, Funding acquisition, Investigation, Methodology, Visualization, Writing – original draft, Writing – review and editing. EN-B: Conceptualization, Data curation, Formal analysis, Investigation, Methodology, Visualization, Writing – review and editing. RA: Data curation, Investigation, Methodology, Writing – review and editing. NA: Methodology, Resources, Writing – review and editing. KJ-S: Conceptualization, Funding acquisition, Project administration, Resources, Writing – review and editing.
