# Supplementary material for: Human iPSC-derived neurons reveal NMDAR-independent dysfunction following HIV-associated insults
Source: Front Mol Neurosci. 2024 Jan 29;16:1353562. doi: 10.3389/fnmol.2023.1353562 (PMC10859444; doi:10.3389/fnmol.2023.1353562)
Supplement: Supplementary file 1 [file Data_Sheet_1.docx]

Supplementary Material

## Supplementary Figures

DAPI

MAP2

MERGE

MDM Mock

MDM HIV-Jago

**B)**

**A)**

**Supplemental Figure S1. Human i^3^ cortical neurons are resistant to cytotoxicity from HIV-Jago-infected myeloid cell supernatants.** A) Representative images of i^3^ neurons following 96-hour exposure to HIV-Jago-MDM supernatants (n=3 donors, mean ± s.e.m.). B) Quantification of average number of MAP2^+^ cells and C) average MAP2^+^ area per cell.

**C)**


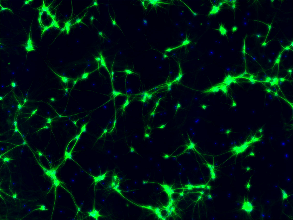

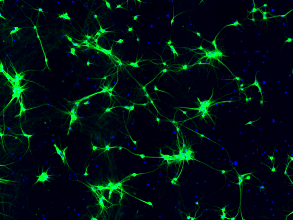

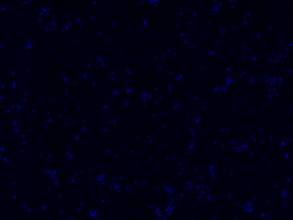

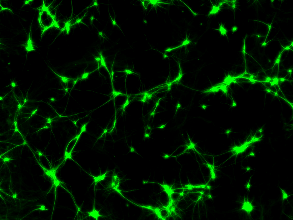

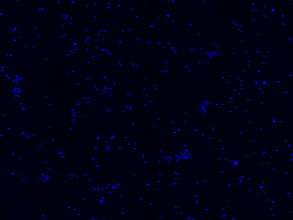

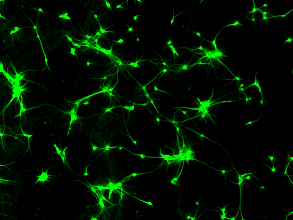

**Supplemental Figure S2. HIV-ADA myeloid cell supernatants do not contain increased glutamate and are not toxic to i^3^ neurons.** A) Glutamic acid concentration in supernatants from HIV-ADA-infected MDMs (n=5 donors ± s.e.m.), and B) iMg (n=3 donors ± s.e.m.) measured by Amplex Red Glutamic Acid Assay Kit (Invitrogen). C) Cytotoxicity in i^3^ neurons treated with HIV-ADA-MDM supernatants (n=3 donors ± s.e.m.) measured by CyQuant LDH Cytotoxicity Assay (Invitrogen).

**C)**

**B)**

**A)**

MERGE

MAP2

DAPI

Veh

**A)**


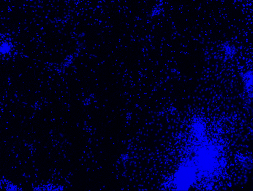

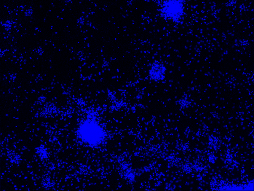

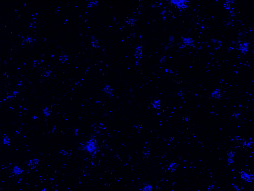

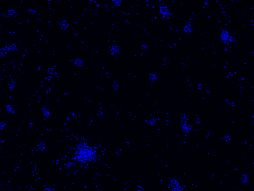

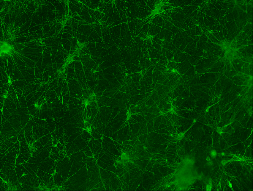

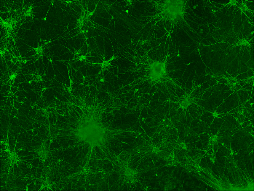

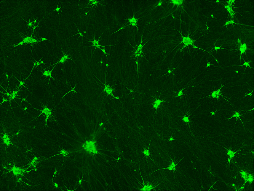

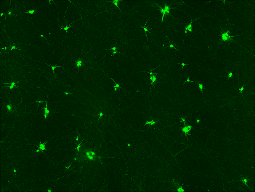

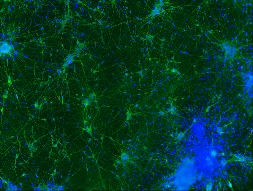

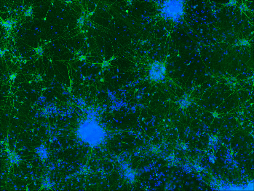

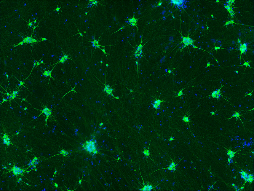

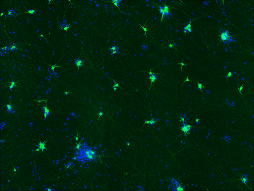


**B)**

1/10 Cmax

3X Cmax

Cmax

**Supplemental Figure S3. High-dose biktarvy is cytotoxic to i^3^ neurons .** A) Representative images of i^3^ neurons following 9 days of exposure to 3 doses of biktarvy (n=3 neuronal differentiations, mean ± s.e.m). B) Quantification of average number of MAP2^+^ cells normalized to untreated.
